# Inhibition of *O*-GlcNAcase Using a Potent and Cell-Permeable Inhibitor Does Not Induce Insulin Resistance in 3T3-L1 Adipocytes

**DOI:** 10.1016/j.chembiol.2010.07.006

**Published:** 2010-09-24

**Authors:** Matthew S. Macauley, Yuan He, Tracey M. Gloster, Keith A. Stubbs, Gideon J. Davies, David J. Vocadlo

**Affiliations:** 1 Department of Chemistry, Simon Fraser University, Burnaby, BC V5A 1S6, Canada; 2 York Structural Biology Laboratory, Department of Chemistry, University of York, Heslington, York YO10 5DD, UK; 3 Department of Molecular Biology and Biochemistry, Simon Fraser University, Burnaby, BC V5A 1S6, Canada

## Abstract

To probe increased *O*-GlcNAc levels as an independent mechanism governing insulin resistance in 3T3-L1 adipocytes, a new class of *O*-GlcNAcase (OGA) inhibitor was studied. 6-Acetamido-6-deoxy-castanospermine (6-Ac-Cas) is a potent inhibitor of OGA. The structure of 6-Ac-Cas bound in the active site of an OGA homolog reveals structural features contributing to its potency. Treatment of 3T3-L1 adipocytes with 6-Ac-Cas increases *O*-GlcNAc levels in a dose-dependent manner. These increases in *O*-GlcNAc levels do not induce insulin resistance functionally, measured using a 2-deoxyglucose (2-DOG) uptake assay, or at the molecular level, determined by evaluating levels of phosphorylated IRS-1 and Akt. These results, and others described, provide a structural blueprint for improved inhibitors and collectively suggest that increased *O*-GlcNAc levels, brought about by inhibition of OGA, does not by itself cause insulin resistance in 3T3-L1 adipocytes.

## Graphical abstract

**Graphical Abstract:**

**Figure:**
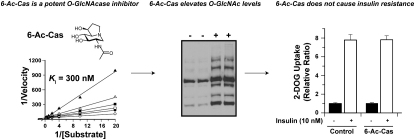

**Highlights:**

► 6-Ac-Cas is a potent inhibitor (*K*_I_ = 300 nM) of human *O*-GlcNAcase ► The structure of an OGA homolog with 6-Ac-Cas reveals the basis for inhibition ► 6-Ac-Cas elevates *O*-GlcNAc levels in cells in a time- and dose-dependent manner ► 6-Ac-Cas does not cause insulin resistance in cultured 3T3-L1 adipocytes

## Introduction

**Figure 1:**
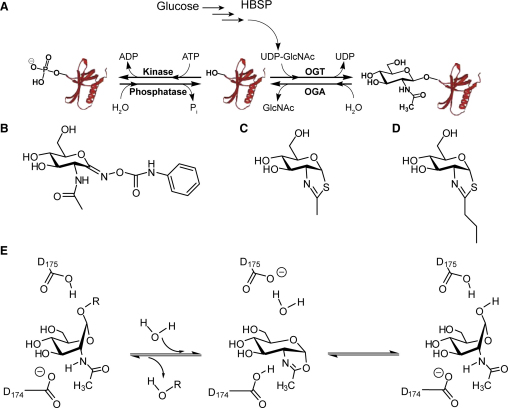
Mechanism of OGA and Commonly Used Inhibitors of OGA (A) The *O*-GlcNAc modification. A percentage of available cellular glucose enters the hexosamine biosynthetic pathway (HBSP) and is converted to UDP-GlcNAc. One glycosyltransferase that uses UDP-GlcNAc as a substrate is the enzyme *O*-GlcNAc transferase (OGT). OGT glycosylates nucleocytoplasmic proteins with β-*O*-linked GlcNAc residues. Conversely, *O*-GlcNAcase (OGA) removes *O*-GlcNAc from modified proteins and imparts a dynamic nature to the *O*-GlcNAc modification. Interplay between this *O*-GlcNAc modification and phosphorylation is thought to give *O*-GlcNAc the ability to modulate cellular signaling. (B–D) The chemical structure of PUGNAc (B), NAG-thiazoline (C), and NButGT (D). (E) OGA uses a mechanism involving substrate-assisted catalysis, which involves the formation of a transient oxazoline intermediate in the first step and subsequent hydrolysis of the intermediate to yield the β-anomer of GlcNAc.

The hexosamine biosynthetic pathway (HBSP) is a metabolic pathway enabling adaptation to variations in concentrations of glucose and other nutrients (Buse, 2006; Marshall, 2006; Teo et al., 2010). Heightened flux through the HBSP leads to the onset of insulin resistance (Marshall et al., 1991) and the precise molecular basis by which this occurs is a topic of great interest since it could lead to clarity regarding the insulin-desensitizing effects of hyperglycemia (van Putten and Krans, 1985). One hypothesis that has emerged is that glucose mediates its effects through increased posttranslational modification of serine and threonine residues of nucleocytoplasmic proteins with a single β-*O*-linked *N*-acetylglucosamine residue (*O*-GlcNAc) (Figure 1A) (Buse, 2006; Hart et al., 2007). The enzyme installing this modification is the glycosyltransferase known as *O*-GlcNAc transferase (OGT). This enzyme uses as a substrate the activated donor sugar uridine disphosphate *N*-acetylglucosamine (UDP-GlcNAc), which is the end product of the HBSP. Because increased glucose availability increases flux through the HBSP and elevates *O*-GlcNAc levels (Robinson et al., 2007; Walgren et al., 2003), the *O*-GlcNAc modification is a candidate nutrient sensing mechanism mediating glucose-induced insulin resistance.

Several studies in cultured cells (Arias et al., 2004; Park et al., 2005; Vosseller et al., 2002) as well as in vivo (Dentin et al., 2008; McClain et al., 2002; Yang et al., 2008) have reported that elevated cellular *O*-GlcNAc levels may be a cause of insulin resistance. These cellular studies have shown that inhibition of *O*-GlcNAcase (OGA) using *O*-(2-acetamido-2-deoxy-d-glucopyranosylidene)amino-*N*-phenylcarbamate (PUGNAc) (Figure 1B) leads to insulin resistance, presumably due to the increased *O*-GlcNAc levels stemming from the continuing action of OGT while OGA function is inhibited (Haltiwanger et al., 1998). PUGNAc has been shown to decrease insulin-stimulated glucose uptake in cultured 3T3-L1 adipocytes (Vosseller et al., 2002; Yang et al., 2008), muscle tissue studied ex vivo (Arias et al., 2004), as well as primary adipocytes (Park et al., 2005); these studies, which all make use of PUGNAc, have provided a key part of the foundation for the hypothesis that increased *O*-GlcNAc levels cause insulin resistance.

A leading hypothesis for a mechanistic link between increased *O*-GlcNAc levels and insulin resistance stems from the observation that *O*-GlcNAc and phosphorylation are reciprocal on certain sites of some proteins (Hart et al., 2007; Wang et al., 2008) (Figure 1A). Therefore, increased *O*-GlcNAc levels have the potential to act as a sensor of excess nutrient availability and provide negative feedback by modulating phosphorylation on key signaling proteins and transcription factors in the insulin signaling cascade (Buse, 2006; Hart et al., 2007). Indeed, several key proteins involved in insulin signaling, such as Akt (Park et al., 2005), IRS-1 (Ball et al., 2006), and GSK-3β (Vosseller et al., 2002), have been found to be *O*-GlcNAc modified and PUGNAc has been shown to hinder their phosphorylation (Vosseller et al., 2002; Whelan et al., 2010; Yang et al., 2008).

PUGNAc is a member of the glyconohydroximolactone family of inhibitors (Dong and Hart, 1994; Horsch et al., 1991) and is commonly used to inhibit OGA in biological settings to study the effects of increased *O*-GlcNAc levels. In addition to OGA, which belongs to family 84 of glycoside hydrolases (GH84) (Cantarel et al., 2009), PUGNAc also inhibits members of GH20 with a similar potency (Gao et al., 2001; Macauley et al., 2005). Consistent with this lack of selectivity, PUGNAc elevates both *O*-GlcNAc levels (Haltiwanger et al., 1998) and levels of the ganglioside GM2 in cultured cells (Ho et al., 2010; Stubbs et al., 2009). PUGNAc also inhibits enzymes using different catalytic mechanisms including β-*N*-acetylglucosaminidases from GH3 (Stubbs et al., 2007) and α-*N*-acetylglucosaminidases from GH89 (Ficko-Blean et al., 2008). A second class of inhibitor that has been used to investigate the function of GH20 and GH84 family members in a biological setting are the thiazoline-based inhibitors (Knapp et al., 1996, 2007; Macauley et al., 2005; Slawson et al., 2008; Tropak et al., 2004; Yuzwa et al., 2008). Both 1,2-dideoxy-2′-methyl-α-d-glucopyranoso-[2,1-d]-Δ2′-thiazoline (NAG-thiazoline) (Figure 1C) and PUGNAc are not selective for GH84 enzymes over GH20 enzymes, whereas 1,2-dideoxy-2′-propyl-α-d-glucopyranoso-[2,1-d]-Δ2′-thiazoline (NButGT) (Figure 1D) is selective for GH84 enzymes including OGA. NButGT and NAG-thiazoline have been proposed to mimic a transition state structurally related to the oxazoline intermediate formed in the active site of enzymes using substrate-assisted catalysis (Knapp et al., 1996; Whitworth et al., 2007) (Figure 1E). Like PUGNAc, NAG-thiazoline also elevates levels of the substrates of both GH20 HexB and GH84 OGA (GM2 and *O*-GlcNAc, respectively) (Stubbs et al., 2009). NButGT, which has two extra methylene units appended to the thiazoline ring (Figure 1D) is 700-fold more potent toward human OGA over human HexB, which belongs to GH20. Consistent with this selectivity, NButGT elevates *O*-GlcNAc levels in cultured cells without increasing levels of GM2 (Stubbs et al., 2009).

Studies making use of NButGT have shown that the off-target effects of PUGNAc may be a concern since NButGT does not recapitulate the insulin-desensitizing effects of PUGNAc in cultured 3T3-L1 adipocytes (Macauley et al., 2008). Studies by other laboratories support this finding; NButGT, unlike PUGNAc, does not hinder activation of Akt in cultured astrocytes (Matthews et al., 2007) and NButGT does not exacerbate high glucose-induced insulin resistance in L6 myotubes (Srinivasan et al., 2009). The surprising absence of any insulin-desensitizing effects arising from inhibition of OGA using the selective inhibitor NButGT may stem from one of two possibilities. The first possibility is that NButGT has secondary off-target effects that reverse the insulin resistance induced by elevated *O*-GlcNAc levels. The second possibility is that increased *O*-GlcNAc levels are not an independent mechanism leading to insulin resistance and it is PUGNAc that has off-target effects that induce insulin resistance. This distinction is an important one since PUGNAc continues to be widely used in studies designed to elucidate the biological roles of *O*-GlcNAc, without concern over its potential off-target effects (Kim et al., 2009; Lee et al., 2010; Nagy et al., 2010; Whelan et al., 2010; Yanagisawa and Yu, 2009).

One way to aid in discriminating which inhibitor has off-target effects, and establish whether increased *O*-GlcNAc levels independently induce insulin resistance in cultured cells, would be to use a structurally distinct inhibitor of OGA. Here, we describe a new OGA inhibitor that has a different structure compared with both PUGNAc and NButGT. We find 6-acetamido-6-deoxy-castanospermine (6-Ac-Cas) elevates *O*-GlcNAc levels but does not cause insulin resistance in 3T3-L1 adipocytes. Further, experiments in which PUGNAc and NButGT or 6-Ac-Cas are used in parallel and in combination indicate these compounds all increase *O*-GlcNAc levels to the same extent yet only PUGNAc induces insulin resistance; these studies also show that neither NButGT nor 6-Ac-Cas reverse insulin resistance induced by PUGNAc. Last, we find that using low doses of PUGNAc, which still increase *O*-GlcNAc levels does not show any insulin-desensitizing effects. These findings collectively support the idea that increased *O*-GlcNAc levels, stemming from inhibition of OGA, are not an independent mechanism governing the development of insulin resistance in 3T3-L1 adipocytes and indicate caution should be used when using PUGNAc in biological settings.

## Results and Discussion

**Figure 2:**
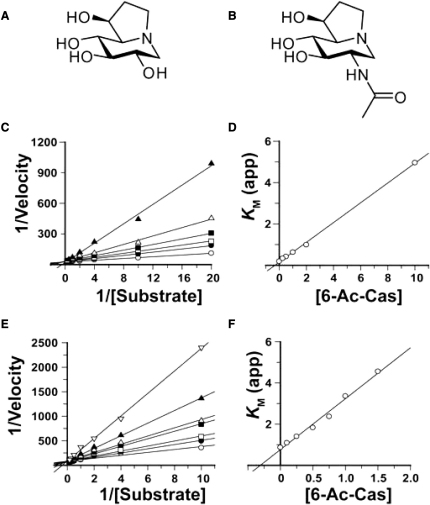
6-Acetamido-6-Deoxy-Castanospermine Is a Potent and Competitive Inhibitor of Both Human OGA and HexB (A) Chemical structure of castanospermine. (B) Chemical structure of 6-acetamido-6-deoxy-castanospermine (6-Ac-Cas). (C and D) Kinetic results for the inhibition of human OGA with 6-Ac-Cas. Velocities are reported as arbitrary absorbance units. (E and F) Kinetic results for the inhibition of human HexB with 6-Ac-Cas. Velocities are reported as arbitrary absorbance units. (C and E) Reciprocal Lineweaver-Burk plots clearly demonstrate the competitive nature of inhibition by 6-Ac-Cas for both enzymes. (D and F) A plot of the apparent *K*_M_ against the concentration of 6-Ac-Cas reveals *K*_I_ values of 300 and 250 nM toward OGA and HexB, respectively.

To clarify whether PUGNAc or NButGT has off-target effects and to establish if increased *O*-GlcNAc levels independently induce insulin resistance in cultured cells, we sought a structurally distinct inhibitor of OGA. One broad class of inhibitors that has proven widely useful for inhibition of many glycoside hydrolases are the iminosugars; these inhibitors have an endocyclic nitrogen in place of the ring oxygen. This class of inhibitor is thought to mimic a dissociative oxocarbenium ion-like transition state structure in which the positive charge is distributed over the anomeric center and the ring oxygen (Lillelund et al., 2002). One member of this class of inhibitor is castanospermine (Figure 2A), a natural product that was originally isolated from *Castanospermum australe* (Hohenschutz et al., 1981). Castanospermine is an iminosugar in which an ethylene unit links the endocyclic nitrogen to create a fused 5,6-ring system, which is typically a low micromolar inhibitor of glucosidases (Gloster et al., 2007). A derivative of castanospermine bearing an acetamido group at the 6-position (corresponding to the 2-position of glucopyranose) has been described as a submicromolar inhibitor of some GH20 family members (Liu et al., 1991; Tropak et al., 2004). This derivative, 6-Ac-Cas (Figure 2B), is structurally different from both the thiazoline-based inhibitors and PUGNAc and is also charged at physiological pH. Given its obvious structural and physical differences, we felt this was a good candidate compound to investigate as an inhibitor of OGA.

### Inhibition of Human OGA and HexB by 6-Ac-Cas

Using 4-nitrophenyl 2-acetamido-2-deoxy-β-d-glucopyranoside (pNP-GlcNAc) as a substrate, the mode of inhibition and *K*_I_ values of 6-Ac-Cas for OGA and HexB were determined (Figures 2C–2F). 6-Ac-Cas acts as a competitive inhibitor toward both enzymes with *K*_I_ values of 300 and 250 nM for OGA (Figures 2C and 2D) and HexB (Figures 2E and 2F), respectively. Although 6-Ac-Cas does not show selectivity for either enzyme, we felt that 6-Ac-Cas would be a good candidate to probe whether NButGT or PUGNAc has off-target effects. Prior to initiating studies using 6-Ac-Cas in cultured cells, we elected to investigate the structural aspects of OGA inhibition by 6-Ac-Cas and its similarities and dissimilarities with NButGT and PUGNAc.

### Structure of *Bt*GH84 in Complex with 6-Ac-Cas

**Figure 3:**
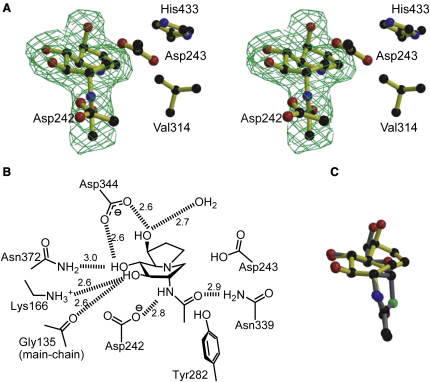
Structure of 6-Ac-Cas Bound in the Active Site of *Bt*GH84 (A) Observed electron density (*F*_obs_-*F*_calc_ “omit” map, contoured at 3σ in divergent stereo) for 6-Ac-Cas bound to *Bt*GH84 (PDB 2XJ7). (B) Schematic diagram showing the interactions between 6-Ac-Cas and *Bt*GH84. All residues shown are conserved in the human GH84 *O*-GlcNAcase. Hydrogen-bond interactions between protein and ligand (≤3 Å) are shown as dashed lines with the distance in Å. (C) Superposition of 6-Ac-Cas in complex with *Bt*GH84 (PDB 2XJ7) with NAG-thiazoline in complex with *Bt*GH84 (PDB 2CHN). For additional details supporting data from this figure, see Table S1 and Figure S1.

A bacterial homolog of human OGA, also belonging to family GH84 of glycoside hydrolases, from *Bacteroides thetaiotaomicron* (*Bt*GH84) was used for structural studies since it has been shown that it is a good model of the human enzyme (Martinez-Fleites et al., 2010). 6-Ac-Cas inhibits *Bt*GH84 with a *K*_I_ value of 220 nM, which closely matched the *K*_d_ obtained using isothermal titration calorimetry (see Figure S1 available online). The structure of *Bt*GH84 cocrystallized with 6-Ac-Cas was solved in the original P1 crystal form (Dennis et al., 2006) at a resolution of 2.0 Å (see Table 1 for data processing and refinement statistics). Unambiguous electron density shows that 6-Ac-Cas binds in the −1 subsite (Davies et al., 1997) as with previous *Bt*GH84 inhibitors (Figure 3A). 6-Ac-Cas is observed in an approximate ^1,4^*B* / ^4^*E* conformation (Vocadlo and Davies, 2008), which is very similar to the conformations reported previously for castanospermine bound to β-glucosidases (Cutfield et al., 1999; Gloster et al., 2007) and reflects distortion of the inhibitor away from the ^4^*C*_1_ chair conformation observed in solution (Hohenschutz et al., 1981). In the retaining β-glucosidase complexes with castanospermine, it is assumed that there is a key interaction between the protonated nitrogen and the nucleophilic carboxylate. Here, consistent with an active site evolved for substrate-assisted catalysis, the acetamido carbonyl oxygen lays ∼2.9 Å “below” the amine moiety of castanospermine, within hydrogen bonding distance (Figure 3B). Beyond this key interaction, 6-Ac-Cas binds similarly to other inhibitors such as NAG-thiazoline (Dennis et al., 2006) and PUGNAc (Macauley et al., 2008). The only difference is a slight change in the position normally occupied by the C6 hydroxyl group, which likely reflects avoidance of a steric clash between Val314 and His433 and the five-membered ring of 6-Ac-Cas (Figure 3C). Overall, this structure highlights how inhibitors with different structures can bind within the enzyme active site in a similar manner. The extensive hydrogen-bonding between 6-Ac-Cas and active site residues is reflected in the highly favorable enthalpy of binding (Δ*H* −8.4 ± 0.5 kcal mol^-1^); there is also a small favorable entropic contribution to binding (TΔ*S* +0.6 kcal mol^-1^) (Figure S1). The 1:1 stoichiometry between *Bt*GH84 and 6-Ac-Cas (Figure S1) is consistent with the observed competitive inhibition derived from kinetics.

### Effect of 6-Ac-Cas on *O*-GlcNAc Levels in 3T3-L1 Adipocytes

**Figure 4:**
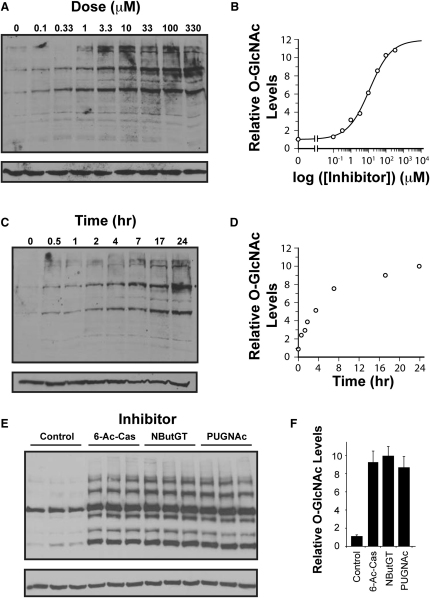
6-Ac-Cas Produces Dose- and Time-Dependent Increases in *O*-GlcNAc-Modified Proteins in 3T3-L1 Cells (A) Fully differentiated 3T3-L1 adipocytes were treated with various doses of 6-Ac-Cas for 24 hr and evaluated for levels of *O*-GlcNAc by western blot. (B) Densitometric analysis of (A) showing the effect of various doses of 6-Ac-Cas on levels of *O*-GlcNAc-modified proteins reveals an *EC*_50_ value of 9 μM. Values shown are reported relative to those found for untreated control cells. (C) Cells treated with 100 μM 6-Ac-Cas display a time-dependent increase in levels of *O*-GlcNAc-modified proteins. (D) Densitometric analysis of (C) showing the effect of 100 μM 6-Ac-Cas on levels of *O*-GlcNAc-modified proteins over time. Values shown are reported relative to those found for untreated control cells. (E) Direct comparison of 3T3-L1 adipocytes treated for 16 hr with 100 μM PUGNAc, NButGT, or 6-Ac-Cas reveals that all three inhibitors elevate levels of *O*-GlcNAc-modified proteins to an equivalent extent over untreated cells. Levels of *O*-GlcNAc-modified proteins were analyzed by western blot analysis using anti-*O*-GlcNAc antibody (CTD110.6) (upper panels) and anti-β-tubulin to evaluate protein loading (lower panels). (F) Densitometric analysis of (E) shows that 100 μM PUGNAc, NButGT, and 6-Ac-Cas all elevate levels of *O*-GlcNAc-modified proteins to the same extent (approximately 10-fold above untreated cells).

To test if 6-Ac-Cas can permeate into cells and inhibit OGA to increase *O*-GlcNAc levels, it was incubated with differentiated 3T3-L1 adipocytes for different periods of times and at different doses of the compound. The 3T3-L1 cell line has been used extensively to study the insulin signaling cascade and the development of insulin resistance; it is also the cell line in which PUGNAc was originally found to induce insulin resistance (Vosseller et al., 2002). As shown in Figure 4A, 6-Ac-Cas elevates *O*-GlcNAc levels in a dose-dependent manner. Densitometric analysis of the complete set of bands in the western blots (see Figure S2 for information on densitometry methods and linear response range for lysates) yields an *EC*_50_ value of 9 μM (Figure 4B), which is consistent with the potency (*K*_I_ = 600 nM) and the *EC*_50_ value of 8 μM that we determined previously for NButGT in this cell line (Macauley et al., 2008). 6-Ac-Cas also elevates *O*-GlcNAc levels in a time-dependent manner (Figures 4C and 4D), with a trend consistent to those observed previously for both NButGT and PUGNAc in this cell line (Macauley et al., 2008). For further studies into the effects of 6-Ac-Cas on insulin sensitivity we chose to use an overnight (16 hr) treatment and a dose of 100 μM, in part to be consistent with previous studies (Macauley et al., 2008; Vosseller et al., 2002) and, in part, because this treatment protocol, with any of the three inhibitors increases *O*-GlcNAc to indistinguishable levels (Figure 4E) that are approximately 10-fold higher than control cells (Figure 4F).

### Effect of 6-Ac-Cas on Insulin-Stimulated 2-DOG in 3T3-L1 Adipocytes

**Figure 5:**
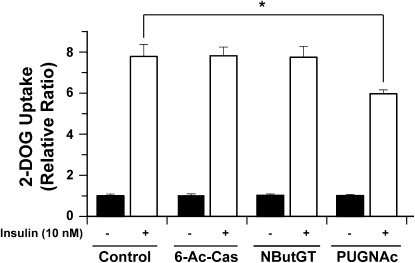
6-Ac-Cas Does Not Affect Insulin-Mediated 2-DOG Uptake in 3T3-L1 Adipocytes 3T3-L1 adipocytes were treated overnight (16 hr) with either 6-Ac-Cas, NButGT, or PUGNAc. The following day, the cells were serum starved for four hours, stimulated with (open bars) or without (closed bars) 10 nM insulin prior to monitoring 2-DOG uptake over a period of 5 min. Values are reported as the relative fold increase in 2-DOG uptake over cells that were not treated with insulin.

To assess the effect of 6-Ac-Cas on insulin sensitivity, 2-deoxyglucose (2-DOG) uptake was determined over 5 min in response to either 0 or 10 nM insulin. Insulin induced an 8-fold increase in 2-DOG uptake compared with control cells. Cells that had been pretreated overnight with 100 μM 6-Ac-Cas took up indistinguishable amounts of 2-DOG compared with control cells (Figure 5). The lack of effect 6-Ac-Cas has on insulin-mediated 2-DOG uptake parallels what is observed in cells treated with NButGT. However, the absence of any insulin-desensitizing effect arising from 6-Ac-Cas treatment, exactly as observed with NButGT treatment, contrasts with the decrease in insulin-stimulated 2-DOG uptake observed when cells are treated with PUGNAc (Figure 5).

### Effect of 6-Ac-Cas on Insulin Signaling in 3T3-L1 Adipocytes

**Figure 6:**
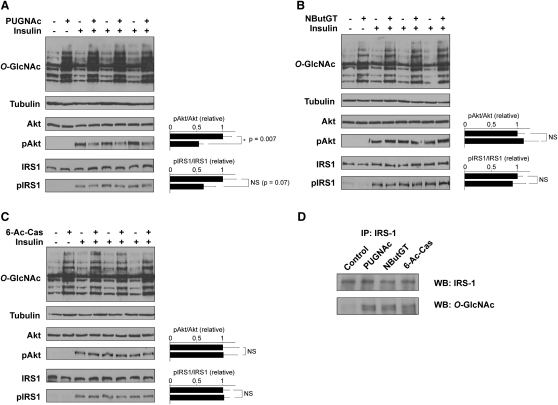
Treatment of 3T3-L1 Adipocytes with 6-Ac-Cas or NButGT Does Not Perturb in Insulin-Mediated Activation of Akt or IRS1 (A–C) Cells were treated with 100 μM (A) PUGNAc, (B) NButGT, or (C) 6-Ac-Cas overnight. The following day, after a 4 hr serum starvation some of the cells were treated with 10 nM insulin. All cells were harvested and analyzed by western blot analysis using the CTD110.6 anti-*O*-GlcNAc, anti-β-tubulin, anti-Akt, anti-pThr308Akt, anti-IRS-1, and anti-pTyr608IRS-1 antibodies. Densitometry of the bands corresponding to pAkt or pIRS-1 relative to total Akt and IRS-1, respectively, are plotted to the right of the blots. (D) IRS-1 was immunoprecipitated from control cells as well as cells treated overnight with PUGNAc, NButGT, or 6-Ac-Cas. The resulting immunoprecipitates were probed by western blot for *O*-GlcNAc and IRS-1. The *O*-GlcNAc levels on IRS-1 from cells treated with each of the three inhibitors are all elevated compared with *O*-GlcNAc levels on IRS-1 from control cells.

PUGNAc has been shown to perturb the insulin signaling pathway by hindering phosphorylation-dependent activation of Akt at Thr308 (Macauley et al., 2008; Vosseller et al., 2002; Yang et al., 2008). A more recent report tracked this effect upstream in the insulin signaling pathway to suggest that it may stem from a defect in phosphorylation of the insulin receptor substrate (IRS-1) (Whelan et al., 2010). Phosphorylation of mouse IRS-1 at Tyr608 (Tyr612 in human IRS-1) (Esposito et al., 2003) and Akt at Thr308 (Sale and Sale, 2008) are both key events in the insulin signaling cascade. Therefore, monitoring insulin-mediated phosphorylation at these residues of IRS-1 and Akt is a useful measure of insulin sensitivity at the molecular level that compliments functional 2-DOG uptake assays. PUGNAc caused a statistically significant 2-fold decrease in insulin-stimulated phosphorylation of Akt at Thr308 (p = 0.007) and a 40% decrease in insulin-stimulated phosphorylation of IRS-1 (p = 0.07) (Figure 6A). On the other hand, neither NButGT nor 6-Ac-Cas perturbed insulin-mediated phosphorylation of either Akt or IRS-1 (Figures 6B and 6C). These findings are consistent with the effects of these three compounds on the 2-DOG uptake. IRS-1 has been demonstrated to be *O*-GlcNAc modified (Park et al., 2005), and the major sites of modification were mapped to the C terminus of the protein (Ball et al., 2006; Klein et al., 2009). To investigate if the three inhibitors increased *O*-GlcNAc modification of IRS-1, IRS-1 was immunoprecipitated from 3T3-L1 adipocytes treated with PUGNAc, NButGT, 6-Ac-Cas, or vehicle alone. As shown in Figure 6D, all three inhibitors increased *O*-GlcNAc levels on IRS-1 to comparable extents, which is in keeping with their use at concentrations well above their respective *EC*_50_ values.

### Effect of Mixing PUGNAc with NButGT on Insulin Resistance in 3T3-L1 Adipocytes

**Figure 7:**
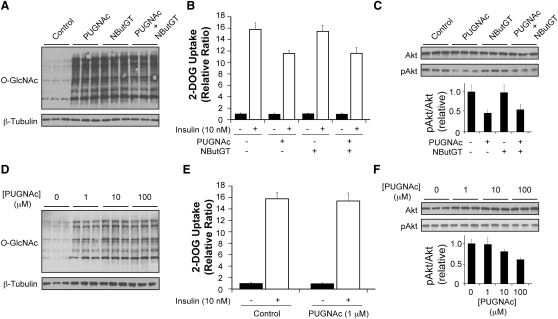
Cotreatment of PUGNAc and NButGT as Well as a Low Dose of PUGNAc Strongly Suggests that Elevated *O*-GlcNAc Levels Do Not Cause Insulin Resistance in 3T3-L1 Adipocytes (A) Assessment of *O*-GlcNAc levels in 3T3-L1 adipocytes treated overnight (16 hr) with either 100 μM PUGNAc, NButGT, or both. (B) 2-DOG uptake assay in 3T3-L1 adipocytes treated overnight with 100 μM PUGNAc, NButGT, or both. Cells were stimulated with (open bars) or without (closed bars) 10 nM insulin. Values are reported as the relative fold increase in 2-DOG uptake over cells that were not treated with insulin. (C) A parallel set of cells were treated identically as in (B) except cells were used for western blot analyses and probed using anti-Akt and anti-pThrAkt antibodies. The lower panel represents densitometry of pAkt levels standardized to total Akt levels. Errors represent the standard deviation between the three independent replicates. (D) Assessment of *O*-GlcNAc levels in 3T3-L1 adipocytes treated overnight (16 hr) with 0, 1, 10, or 100 μM PUGNAc. (E) 2-DOG uptake assay in 3T3-L1 adipocytes treated overnight with or without 1 μM PUGNAc. Cells were stimulated with (open bars) or without (closed bars) 10 nM insulin. Values are reported as the relative fold increase in 2-DOG uptake over cells that were not treated with insulin. (F) Cells treated with 0, 1, 10, or 100 μM PUGNAc were stimulated with 10 nM insulin as in (E) except cells were used for western blot analyses and probed using anti-Akt and anti-pThrAkt antibodies. The lower panel represents densitometry of pAkt levels standardized to total Akt levels. Errors represent the standard deviation between the three independent replicates. For additional experiments supporting data from this figure, see Figure S3.

3T3-L1 adipocytes were treated with PUGNAc or NButGT or both together in order to test if NButGT has an effect that mitigates insulin resistance. Cotreatment of the two inhibitors resulted in the same increase in *O*-GlcNAc levels compared with treatment of either inhibitor alone (Figure 7A). Cotreatment of PUGNAc and NButGT resulted in the same 25% decrease in 2-DOG uptake that is observed when cells are treated with PUGNAc alone (Figure 7B). The results at the molecular level agree with this finding; cotreatment did not reverse the defect in activation of Akt compared with cells treated with PUGNAc alone (Figure 7C). The same results on the activation of Akt were observed with cotreatment of cells with PUGNAc and 6-Ac-Cas (Figure S3). These results strongly suggest that NButGT and 6-Ac-Cas do not have off-target effects that can reverse insulin resistance.

### Effect of Using a Low Dose of PUGNAc on Insulin Resistance in 3T3-L1 Adipocytes

PUGNAc is approximately 12 times more potent than NButGT under physiological conditions (pH 7.4) (Macauley et al., 2005; Yuzwa et al., 2008). Consistent with this, we previously demonstrated that PUGNAc has a lower *EC*_50_ value than NButGT in 3T3-L1 adipocytes (Macauley et al., 2008). The studies that have shown PUGNAc causes insulin resistance (Arias et al., 2004; Vosseller et al., 2002; Yang et al., 2008), including our previous study (Macauley et al., 2008) and the results presented above, have used PUGNAc at a concentration of 100 μM or higher. These concentrations are significantly above the 3 μM *EC*_50_ value for PUGNAc and so we assessed the effect of PUGNAc on insulin sensitivity of 3T3-L1 adipocytes at a lower concentration closer to the *EC*_50_ value yet still well above the *K*_I_ value. At 1 μM, PUGNAc still caused a dramatic elevation of *O*-GlcNAc levels. These levels were maximal at 10 μM PUGNAc since *O*-GlcNAc levels were indistinguishable between cells treated with 10 and 100 μM PUGNAc (Figure 7D). Despite the increased *O*-GlcNAc levels present in cells treated with 1 μM PUGNAc, this treatment regimen did not blunt insulin-stimulated 2-DOG glucose uptake (Figure 7E). Consistent with this observation, 1 μM PUGNAc did not perturb insulin-stimulated activation of Akt (Figure 7F). At 10 μM PUGNAc, there was only a small and statistically insignificant (p > 0.05) decrease in insulin-stimulated pAkt levels (Figure 7F). These results contrast with the insulin-desensitizing effect produced by 100 μM PUGNAc despite the indistinguishable differences in the levels of *O*-GlcNAc in cells treated with 10 versus 100 μM PUGNAc.

Together, the studies presented here using 6-Ac-Cas, the cotreatment of cells with PUGNAc and NButGT or 6-Ac-Cas, and the low concentration studies using PUGNAc, all suggest that the insulin-desensitizing effect of PUGNAc is not due to inhibition of OGA leading to increased *O*-GlcNAc levels. Interestingly, ganglioside levels have been proposed to impact insulin signaling (Kabayama et al., 2005). As discussed, PUGNAc and 6-Ac-CAS both inhibit OGA as well as the lysosomal GH20 β-hexosaminidases responsible for degradation of gangliosides. The observation that 6-Ac-CAS does not induce insulin resistance in 3T3-L1 adipocytes is therefore notable because it suggests that inhibition of these lysosomal β-hexosaminidases is not the mechanism by which PUGNAc induces insulin resistance. It is possible to entertain various other scenarios as to the off-target effects of PUGNAc that induce insulin resistance. Because PUGNAc has an aromatic aglycon one scenario might be that it could act as a substrate decoy for cellular glycosyl transferases (Lugemwa and Esko, 1991), which could lead to perturbations in cell surface glycosylation. Alternatively, PUGNAc could act as an inhibitor of glucose transport. Such a scenario is unlikely to be the mechanism since molecular effects on the insulin signaling pathway are observed with PUGNAc treatment. One intriguing possibility is that the oxime carbamate moiety of PUGNAc may be reactive enough to serve as an acylating agent. This possibility is supported by the observation that compounds related to PUGNAc can break down in aqueous solution (Macauley et al., 2005) and the noted requirement that addition of inhibitor to the media is needed for extend periods of time when treating cells (Haltiwanger et al., 1998; Slawson et al., 2005). Indeed, oxime carbamates have been used as activity-based proteomics profiling probes (Li et al., 2007). Though PUGNAc does have off-target effects, the basis of these effects may prove to be an interesting tangential line of research leading to new targets in the insulin signaling pathway.

The results we describe showing that increased *O*-GlcNAc does not on its own lead to insulin resistance find conceptual support from a study that showed that decreasing *O*-GlcNAc levels in 3T3-L1 adipocytes, through several genetic approaches, did not reverse insulin resistance caused by culturing cells in high glucose (Robinson et al., 2007). It is important to note that in spite of these findings in cultured cells, a number of other studies have observed signs of insulin resistance or perturbed glucohomeostasis in vivo when OGT is overexpressed (Dentin et al., 2008; Housley et al., 2008; McClain et al., 2002; Yang et al., 2008). Differences between pharmacological and genetic methods used to increase *O*-GlcNAc levels could be one explanation (Knight and Shokat, 2007). Alternatively, because several of these genetic studies have been carried out in vivo it is possible that it is only in the complex setting found within live animals that insulin resistance may develop. To date, however, no studies have investigated the effects of pharmacologically elevated *O*-GlcNAc levels on glucohomeostasis in vivo. In the next article (Macauley et al., 2010) (this issue of *Chemistry* & *Biology*), we describe the use of NButGT in vivo and use various experimental paradigms to test the effects that increased *O*-GlcNAc levels have on glucohomeostasis.

## Significance

**The discovery of OGA inhibitors has made critical contributions to the field of O-GlcNAc biology, enabling evaluation and generation of hypotheses as to the functional roles of *O*-GlcNAc in cells. Earlier inhibitors such as streptozotocin have been shown, through elegant studies, to have clear off-target effects (****Gao et al., 2000; Pathak et al., 2008****). Like streptozotocin, PUGNAc has been widely used and has been important for establishing the dynamic nature of *O*-GlcNAc and for providing key support for the nutrient sensing hypothesis. Despite concerns about its selectivity, PUGNAc continues to be used to study the function of *O*-GlcNAc. To clarify whether newer selective inhibitors such as NButGT have off-target effects, or, alternatively, whether it is PUGNAc that has off-target effects, we have sought new structurally distinct inhibitors of OGA. Here, we describe 6-Ac-Cas as a class of OGA inhibitor, distinct from both PUGNAc and NButGT, which acts efficiently in cells to increase *O*-GlcNAc levels in a dose- and time-dependent manner. The co-crystal structure of *Bt*GH84 in complex with 6-Ac-Cas reveals the molecular basis for inhibition of OGA. This structure presents a blueprint that should enable the future design of derivatives based on this scaffold that are selective for either OGA or HexB. The concordance of results showing that neither 6-Ac-Cas nor NButGT cause insulin resistance in 3T3-L1 adipocytes strongly points to the insulin-desensitizing effects of PUGNAc arising from off-target effects. These data suggest that caution should be used when interpreting data obtained using PUGNAc in a biological setting. Overall, our studies suggest that pharmacological elevation of *O*-GlcNAc levels do not induce insulin resistance in 3T3-L1 adipocytes. The results should stimulate further study into the inhibition of OGA in vivo and open the way for using different classes of inhibitors to probe *O*-GlcNAc biology.**

## Experimental Procedures

### General

NButGT was prepared as described previously (Macauley et al., 2005). PUGNAc and 6-Ac-Cas were obtained from Toronto Research Chemicals and Industrial Research Limited, respectively. pNP-GlcNAc was obtained from Sigma. Dexamethasone and isobutylmethylxanthine were obtained from Sigma. Insulin was obtained from Eli Lilly. Human OGA was recombinantly expressed as described previously (Cetinbas et al., 2006). Human HexB was obtained from Sigma.

### Inhibition by 6-Ac-Cas Human OGA and HexB

To determine the inhibition constant of 6-Ac-Cas for OGA, a continuous UV/Vis assay was carried out using 4-nitrophenyl 2-acetamido-2-deoxy-β-d-glucopyranoside as a substrate. The substrate was varied between 50 μM to 3.5 mM and inhibitor concentration was varied between 50 nM to 2 μM. The apparent *K*_M_ at each inhibitor concentration was determined and plotted against the inhibitor concentration. In this manner, the *K*_I_ could be calculated as the negative of the value where the best-fit line crossed the x axis. The buffer conditions and precise details of how the assay was carried out have been described previously (Cetinbas et al., 2006).

### *Bt*GH84 Structure Solution and Refinement

The complex of *Bt*GH84 with 6-Ac-Cas was obtained by soaking *apo* crystals, which were obtained as described previously (Dennis et al., 2006), with a small quantity of powdered 6-Ac-Cas. Crystals were obtained in space group P1 with cell dimensions *a* = 51.2 Å, *b* = 92.7 Å, *c* = 98.8 Å, α = 103.0°, β = 95.0°, and γ = 101.3° and with two molecules of *Bt*GH84 in the asymmetric unit. X-ray diffraction data were collected at the European Synchrotron Radiation Facility (ESRF, Grenoble) on beam-line ID23-1 to 2.0 Å resolution (details in Table 1), and data were processed with MOSFLM (Leslie, 1992). All other crystallographic computing used the CCP4 suite unless otherwise stated. The structure was refined, using the original *apo* structure (Dennis et al., 2006) as the starting model, with REFMAC (Murshudov et al., 1997) and model building and addition/inspection of solvent molecules and inhibitor was performed using COOT (Emsley and Cowtan, 2004). Electron density figures were prepared with BOBSCRIPT (Esnouf, 1997).

### Cell Culture

3T3-L1 preadipocytes were obtained from Dr. Green at Harvard Medical School. 3T3-L1 adipocytes were cultured as preadipocytes and then differentiated into adipocytes using a previously reported protocol (Macauley et al., 2008). For 2-DOG uptake assays, cells were differentiated in 12-well plates, whereas for experiments aimed at assessing the phosphorylation of IRS-1 and Akt, the cells were differentiated in 6-well plates. All assays with 3T3-L1 adipocytes took place 10–12 days after differentiation; a time in which >95% of cells displayed adipocyte morphology.

### 2-DOG Uptake

Fully differentiated 3T3-L1 adipocytes were treated overnight (16 hr) with the appropriate dose of inhibitor. The following day, the media was removed, cells were washed once with a large volume of PBS, DMEM containing low glucose (5 mM) without serum was added and cells were incubated for 4 hr. During this time, inhibitors were supplemented at the same concentration as treated overnight. This media was then removed, cells were washed twice with PBS, and cells were incubated in Krebs-Ringer Phosphate (KRP) buffer. After 15 min, insulin was added to some wells to a final concentration of 10 nM. After another 15 min, [1-^3^H] 2-DOG (0.5 μCi/ml, 100 μM) (Moravek Radiochemicals) was added to the cells and after precisely 5 min the assay was terminated. To ensure that assays were stopped at the same time, the liquid in the plates was dumped into a discard bucket and the plates were submerged in 1 liter of cold PBS. The plate was then submerged into a second fresh bucket of PBS, then dried on paper towel, and 500 μl of Triton X-100 was added to each well. After thorough homogenization of the contents in each well by pipetting the contents up and down approximately ten times, 300 μl was used for scintillation counting to determine the amount of 2-DOG taken up into cells.

### Western Blotting

The assay was identical to the 2-DOG uptake assay except, following the 15 min of insulin stimulation, the contents of the cells were removed and 300 μl of 1× SDS-PAGE loading buffer was added to each well. The contents of each well were carefully transferred into a conical tube and heated at 95°C for 15 min. The lysates were directly used for western blotting using procedures outlined previously (Macauley et al., 2008) with the only modification being that for blots toward Akt, pAkt, IRS-1, or pIRS-1 5% nonfat milk powder in PBS containing 0.1% Tween 20 (PBS-T) was used to initially block the nitrocellulose membrane. For western blot analysis, between 10 and 50 μg of protein from lysates was used to load each lane. Exposure times of films to nitrocellulose membranes ranged from between 5 and 30 s. For the Akt and pAkt blots, shorter washing times (15 min total compared with 1 hr total) after the primary and secondary antibodies was used. The Akt and pAkt antibodies were obtained from Cellular Signaling Technologies and used at a dilution of 1:1000. The anti-IRS-1 antibody used for western blotting was obtained from Santa Cruz Biotechnology and used at a dilution of 1:2000, while the pIRS-1 antibody was obtained from Upstate and used at a dilution of 1:2000. The anti-IRS-1 antibody used for immunoprecipitations was obtained from Upstate. The RL2 anti-*O*-GlcNAc antibody was obtained from Abcam and used at a dilution of 1:1000. The CTD110.6 anti-*O*-GlcNAc antibody was obtained from Covance and used at a dilution of 1:4000. The anti-β-tubulin antibody was obtained from the Developmental Studies Hybridoma Bank and used at a dilution of 1:2500. All secondary antibodies were HRP-conjugated, obtained from Santa Cruz Biotechnology, and used at a dilution of 1:20,000.

### Preparation of 3T3-L1 Lysates for Immunoprecipitation

Differentiated 3T3-L1 adipocytes in 10 cm culture dishes were treated with inhibitors overnight (16 hr). The following day, the media was removed and cells were washed with 5 ml of PBS. Cells were then gently scrapped off the plate in 10 ml of PBS and pelleted by centrifugation (10 min, 250 rcf). The supernatant was carefully decanted and the pellets were stored at −80°C. The pellets were thawed and resuspended in 0.5 ml of PBS containing 0.5% nonidet P-40 (Ipegal) along with protease inhibitors (Roche) and 1 mM NButGT. The pellets were passed through a 27 gauge needle three times, rocked for 30 min at 4°C, and sonicated twice for 20 s. The solutions were centrifuged (10 min, 17,000 rcf) to remove insoluble debris and the supernatant was gently removed and set aside. To this supernatant, an equal volume (0.5 ml) of 1,1,1-trichloro-2,2,2,-trifluoroethane was added, and the mixture was inverted approximately ten times, in order to sequester lipids. This solution was centrifuged (10 min, 17,000 rcf) and the top layer was removed and used in the immunoprecipitations.

### Immunoprecipitation of IRS-1

Lysates from 3T3-L1 adipocytes, prepared using the protocol described above, were incubated with 10 μl of anti-IRS-1 antibody. The solutions were gently rocked at 4°C for 2 hr. Protein A/G beads (40 μl, Calbiochem) were then added to capture the primary antibody and these mixtures were further rocked at 4°C for an hour. The beads were centrifuged (30 s, 2000 rcf) and the supernatant was gently removed. The beads were washed with 1 ml of cold lysis buffer by inverting the tube ten times. Following another spin and removal of this supernatant, the washing procedures were repeated once more with lysis buffer and then once with cold PBS for a total of three washes. After the final wash, after which the beads were sucked dry and 80 μl of 1× SDS-PAGE loading buffer was added, the beads were heated at 95°C for 15 min, and then finally centrifuged (30 s, 2000 rcf). The supernatant was gently removed and set aside to use for western blots.

## Figures and Tables

**Table 1:** Data Collection and Structure Refinement Statistics for the Complex of *Bt*GH84 with 6-Ac-Cas

	*Bt*GH84 - 6-Ac-Cas
**Data Collection**	

Space group	P1
Cell dimensions	
*a*, *b*, *c* (Å)	51.5, 94.0, 98.8
α, β, γ (°)	104.1, 93.9, 103.1
Resolution (Å)	50.00–2.00 (2.07–2.00)
R_merge_	0.054 (0.452)
*I* / σ*I*	15.4 (2.0)
Completeness (%)	98 (97)
Redundancy	2.4 (2.4)

**Refinement**	

Resolution (Å)	2.0
No. reflections	108,834
R_work_ / R_free_	0.20/0.24
No. atoms	
Protein	10,398
Ligand/ion	32
Water	394
*B*-factors^a^	
Protein	44
Ligand/ion	30
Water	42
Rmsds	
Bond lengths (Å)	0.020
Bond angles (°)	1.59

a Atomic *B*-values include the TLS contribution.

## Accession Numbers

Coordinates have been deposited in the Protein Data Bank (PDB) with accession code 2XJ7.

## Supplemental Information

Document S1. One Table, Three Figures, and Supplemental Experimental Procedures
