# Inhibition of *O*-GlcNAcase Using a Potent and Cell-Permeable Inhibitor Does Not Induce Insulin Resistance in 3T3-L1 Adipocytes

**DOI:** 10.1016/j.chembiol.2010.10.006

**Published:** 2010-10-29

**Authors:** M.S. Macauley, Y. He, T.M. Gloster, K.A. Stubbs, G.J. Davies, D.J. Vocadlo

(Chemistry & Biology *17*, 937–948; September 24, 2010)

The following conflict of interest statement was inadvertently omitted from the final manuscript during the production process:

M.S.M., K.A.S., and D.J.V. are eligible to receive royalties from Simon Fraser University. G.J.D. is a member of the scientific advisory board of Alectos Therapeutics. D.J.V. is a founder, shareholder, consultant, and member of the scientific advisory board of Alectos Therapeutics.

*Chemistry & Biology* apologizes for this omission.

