# Supplementary material for: Inhibition of O-GlcNAcase Using a Potent and Cell-Permeable Inhibitor Does Not Induce Insulin Resistance in 3T3-L1 Adipocytes
Source: Chem Biol. 2010 Sep 24;17(9):937–48. doi: 10.1016/j.chembiol.2010.07.006 (PMC2954295; doi:10.1016/j.chembiol.2010.07.006)
Supplement: Document S1. One Table, Three Figures, and Supplemental Experimental Procedures [file mmc1.pdf]

Chemistry & Biology *17*

Supplemental Information

Inhibition of *O*-GlcNAcase Using a Potent and Cell-Permeable Inhibitor Does Not Induce Insulin Resistance in 3T3-L1 Adipocytes

Matthew S. Macauley, Yuan He, Tracey M. Gloster, Keith A. Stubbs, Gideon J. Davies, and David J. Vocadlo

Table S1. Data collection and structure refinement statistics for the complex of *Bt*GH84 with 6-Ac-Cas

	**B*t*GH84 - 6-Ac-CAS**
**Data collection**	
Space group	P1
Cell dimensions	
a, *b*, *c* (Å)	51.5, 94.0, 98.8
α, β, γ (°)	104.1, 93.9, 103.1
Resolution (Å)	50.00 - 2.00 (2.07 - 2.00)
R_{merge}	0.054 (0.452)
$I / \sigma I$	15.4 (2.0)
Completeness (%)	98 (97)
Redundancy	2.4 (2.4)
**Refinement**	
Resolution (Å)	2.0
No. reflections	108834
R_{work} / R_{free}	0.20 / 0.24
No. atoms	
Protein	10398
Ligand/ion	32
Water	394
B-factors*	
Protein	44
Ligand/ion	30
Water	42
R.m.s. deviations	
Bond lengths (Å)	0.020
Bond angles (°)	1.59

* Atomic *B*-values include the TLS contribution.

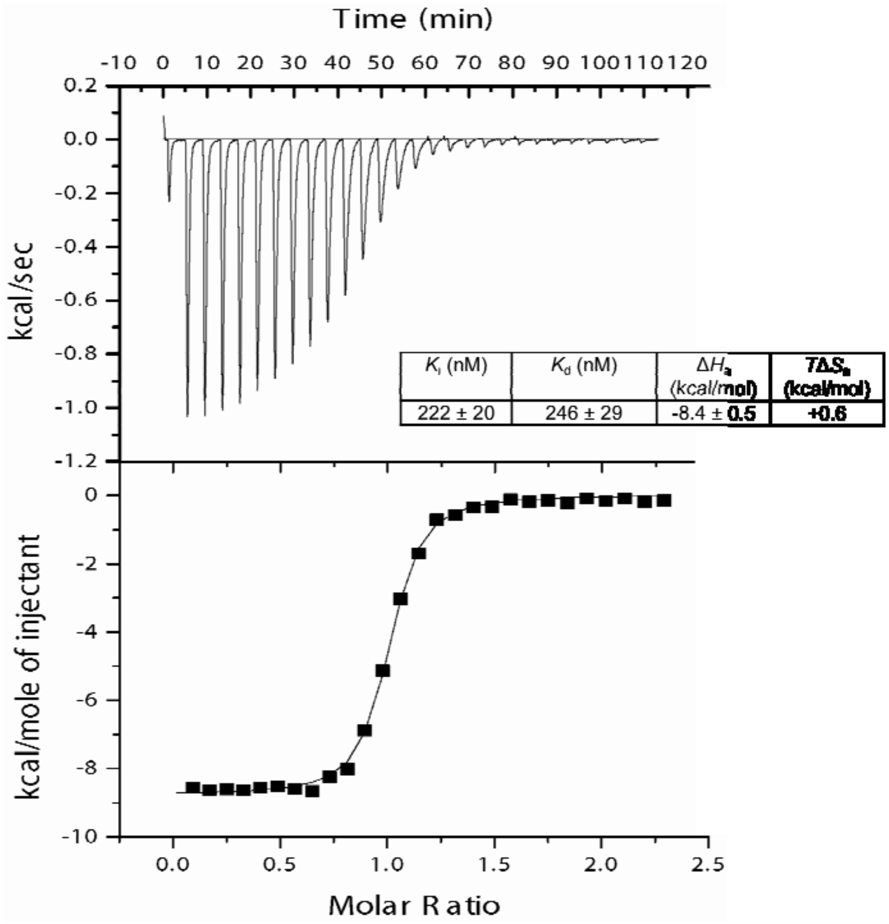

K_i (nM)	K_d (nM)	ΔH_a (kcal/mol)	$T\Delta S_a$ (kcal/mol)
222 ± 20	246 ± 29	-8.4 ± 0.5	+0.6

Figure S1, related to Figure 3. Isothermal titration calorimetry plot for *Bt*GH84 with 6-Ac-Cas

The upper panel shows the power supplied to the system to maintain a constant temperature against time (the area of each peak gives the heat of interaction for that injection). The lower panel shows the bimolecular fit of the normalised heats of interaction plotted against the molar concentration. Assays were carried out in 50 mM MES, pH 6.5, 200 mM NaCl at 25 °C. The data of K_d and ΔH_a are average values from duplicate runs.

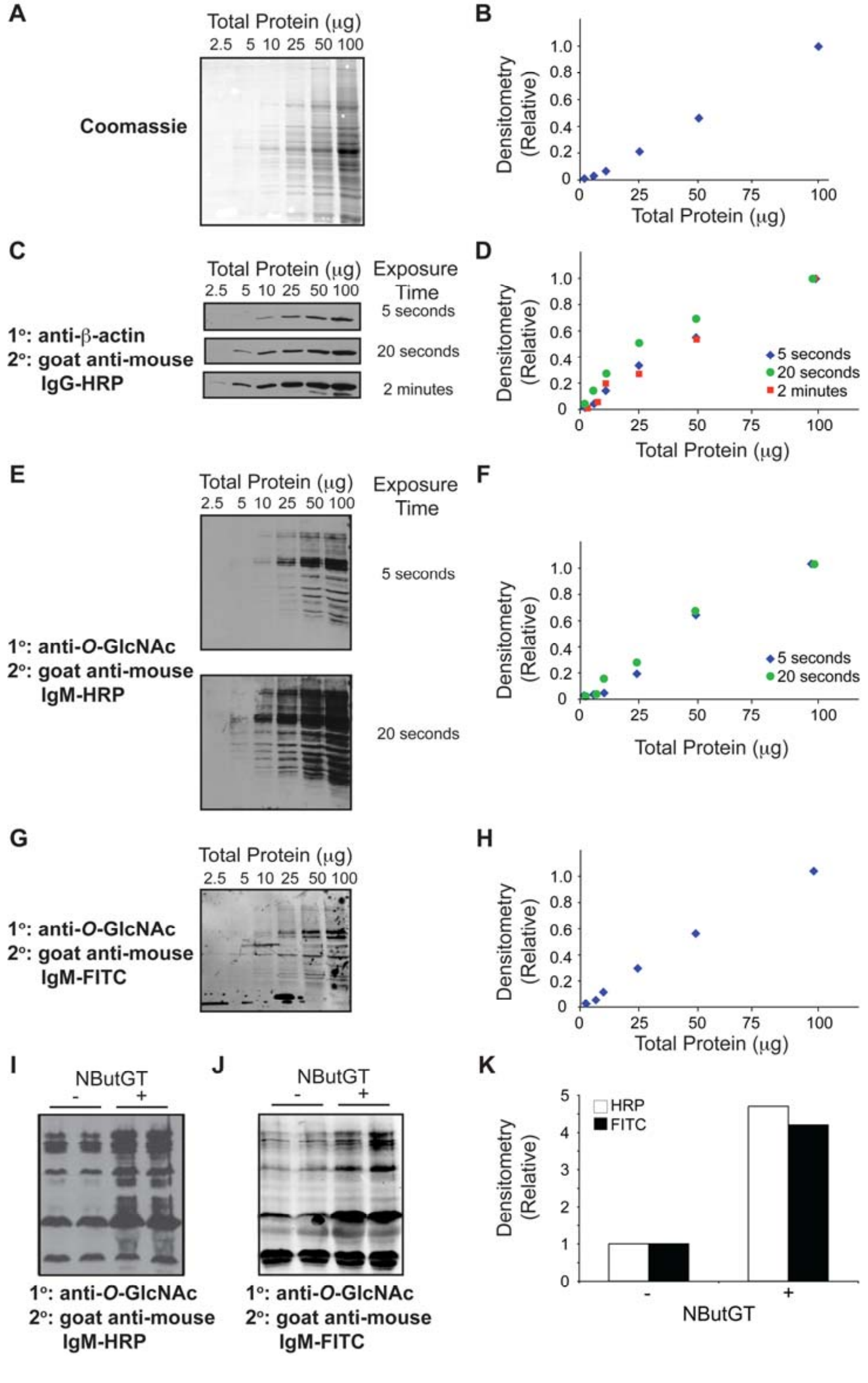

Figure S2. Quantitation of western blot analyses of lysates by densitometry through the generation of standard curves and comparison of film with fluoresence imaging
(A) Varying amounts of brain tissue lysate were loaded onto a 10% SDS-PAGE gel and electrophoresed. The gel was stained with Coomassie blue G-250, destained, and then scanned.
(B) Densitometry of the entire lane for each quantity of protein loaded in panel A. Densitometric measurements are linear in the range of 5 – 100 μg of total protein.

(C) Three gels were run that were loaded identically to that in panel A. Proteins from these gels were transferred to nitrocellulose membrane using a wet protocol and probed for β-actin using an anti-β-actin antibody, followed by goat anti-mouse IgG secondary antibody conjugated to horseradish peroxidase (HRP). Following washing and addition of the HRP substrate, the blots were exposed to film for different times as indicated. These three films were then scanned and analyzed by densitometry for quantitation.
(D) Densitometry of the band corresponding to β-actin for each quantity of protein loaded in panel C. Each exposure length is plotted as different symbols; 5 seconds (blue diamonds); 20 seconds (green circles); and 2 minutes (red squares). Densitometric measurements are approximately linear in the range of 5 – 50 μg of total protein, however, linearity broke down faster at high protein amounts with a longer exposure time.
(E) Two gels were loaded identically to that in panel A and electrophoresed. Proteins from these gels were transferred to nitrocellulose membrane using a wet protocol and probed for *O*-GlcNAc using an anti-*O*-GlcNAc antibody (CTD110.6) followed, after washing, by goat anti-mouse IgM secondary antibody conjugated to horseradish peroxidase (HRP). Following addition of the HRP substrate, the blots were exposed to film to different times as indicated. These three films were then scanned and analyzed by densitometry for quantitation.
(F) Densitometry of the entire lane for each quantity of protein loaded in panel E. Each exposure length is plotted as different symbols: 5 seconds (blue diamonds); 20 seconds (green circles). Densitometric measurements are approximately linear in the range of 5 – 50 μg of total protein, however, linearity breaks down faster at low protein amounts with a shorter exposure time.
(G) A gel was loaded identically to that in panel A and electrophoresed. In the same manner as described above in E, the blot was probed for *O*-GlcNAc except this time a fluorescently labeled secondary antibody was used. In this case, a Typhoon imager was used for visualization and quantitation.
(H) Densitometry of entire lane for each quantity of protein loaded in panel G. Densitometric measurements are linear over a range of 2.5 – 50 μg of total protein. Consistent with the known fact that X-ray film has limited range of linear response, these results with a fluorescently-labeled secondary antibody are linear over a broader range, in particular with high amounts of loaded protein. Note however, that the use of the fluorescently-labeled secondary is much less sensitive.
(I, J) Comparison in quantification of *O*-GlcNAc levels between NButGT-treated samples using an HRP- or fluorescently-labeled secondary antibody. 10 μg of pancreatic tissue extract from animals treated with (+) or without (-) NButGT were loaded and used in Western blot with an HRP- (I) or fluorescently- (J) labeled secondary antibody. Blots were developed/visualized by exposure to either film or a Typhoon imager, respectively.
(K) Densitometry of entire lane for each condition. Numbers represent the average between the two replicates. Similar vales for the increase in *O*-GlcNAc levels (≈ 4.5-fold in this case) were obtained using either visualization method.

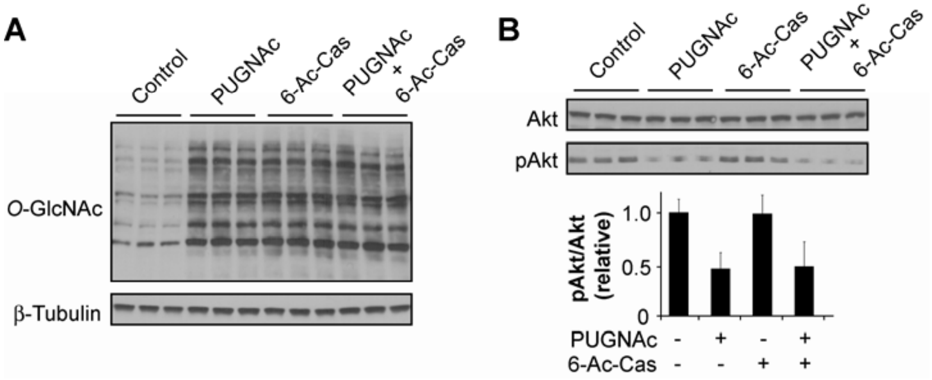

Figure S3, related to Figure 7. Co-treatment of PUGNAc and 6-Ac-Cas does not cause a reversal of insulin resistance in 3T3-L1 adipocytes
(A) Assessment of *O*-GlcNAc levels in 3T3-L1 adipocytes treated overnight (16 hr) with either 100 μM PUGNAc, 6-Ac-Cas, or both inhibitors. At cells were stimulated with 10 nM insulin for 15 minutes prior to harvesting.
(B) The same cells used in (A) were also used for Western blot analyses with an anti-Akt and anti-pThrAkt antibody. The lower panel represents densitometry of pAkt levels standardized to total Akt levels. Errors represent the standard deviation between the three independent replicates.

SUPPLEMENTAL EXPERIMENTAL PROCEDURES

BtGH84 Kinetics - Kinetic studies were conducted by monitoring the change in UV-Visible absorbance at 400 nm using a Cintra 10 spectrophotometer. Assays were carried out at 25°C in thermally equilibrated disposable cuvettes, containing a total volume of 1 ml buffer (50 mM MES, pH 6.5, 200 mM NaCl) with 50 μM 4-nitrophenyl 2-acetamido-2-deoxy-β-D-glucopyranoside (pNP-GlcNAc) as substrate. Reactions were initiated by the addition of 10 μL of 1.9 μM *Bt*GH84 using a syringe. 6-Ac-Cas concentrations in the assay varied from 200 nM to 1 μM. 4-Nitrophenolate release was recorded continuously for 300 seconds. Rates in both the presence and absence of inhibitor were determined as the slope of the linear region over a 100 second period. The K_i (inhibition constant) value was determined using the equation:

$$\frac{v_o}{v_i} = \frac{1}{K_i}.[I] + 1$$

where ν_o and ν_i are the rates of reaction in the absence and presence of inhibitor, respectively. A plot of v_0/v_i against increased inhibitor concentrations yields a gradient of $1/K_i$, with an intercept of 1.

Isothermal Titration Calorimetry (ITC) with BtGH84 - ITC measurements were performed using a MicroCal VC calorimeter (Northampton, MA). All measurements were carried out in 50 mM MES, pH 6.5 and 200 mM NaCl at 25°C. Purified *Bt*GH84 protein was extensively dialyzed against the above buffer, and the dialyzate was subsequently used to dilute the 6-Ac-Cas. The enzyme and ligand concentrations used in

these experiments were 34-50 μM and 0.5 mM, respectively. All samples were centrifuged and degassed prior to use. For each titration, 10 μL aliquots of the inhibitor were injected into *Bt*GH84 in the cell, at an interval of 4 min, with 307 rpm stirring speed. The experimental data were fitted to a non-linear regression model using Microcal Origin software, with stoichiometry (n), enthalpy ($\Delta H°$) and association constant (K_a) as adjustable parameters. The thermodynamic parameters $\Delta G°$ (free energy) and $\Delta S°$ (entropy) were derived from the standard equation: RT $\ln(K_a) = \Delta G° = \Delta H° - T\Delta S°$.
